# Goblet Cell Density of Adhesive Structures Correlates With Climbing Ability in Hawaiian Stream Gobies

**DOI:** 10.1002/jmor.70078

**Published:** 2025-08-26

**Authors:** Amanda M. Palecek‐McClung, Charles H. Christen, Dharamdeep Jain, Ali Dhinojwala, Richard W. Blob, Heiko L. Schoenfuss

**Affiliations:** ^1^ Department of Biological Sciences Clemson University Clemson South Carolina USA; ^2^ Aquatic Toxicology Laboratory St. Cloud State University St. Cloud Minnesota USA; ^3^ Department of Polymer Science, Integrated Bioscience Program The University of Akron Akron Ohio USA

**Keywords:** adhesion, attachment, fish, mucus, spectroscopy

## Abstract

Hawaiian stream gobies exhibit diverse adhesive abilities that can be used by these fishes to help climb waterfalls. Mucus is recognized as contributing to successful performance in many adhesive systems, but potential specializations of mucus production and composition have not been tested in these fishes. This study examines how anatomical (sucker size and goblet cell density) and biochemical (mucus composition) traits may contribute to adhesive success in climbing gobies. Using histological and spectroscopic analyses, we quantified the density of mucus‐producing goblet cells in adhesive structures (lips and pelvic suckers) and assessed differences in mucus chemistry between the pelvic suckers and the body. Goblet cell density in lips and suckers increased with climbing ability, aligning with species distribution across stream elevations. The non‐climbing *Stenogobius hawaiiensis* exhibited the lowest goblet cell densities, while the best climbers (*Sicyopterus stimpsoni* and *Lentipes concolor*) showed the highest densities. Among inching climbing gobies that use the mouth to climb especially as juveniles, goblet cell density in the lips was, instead, greater in adult individuals. This suggests that mucus production of the lips may have a broader protective role during interactions with rough substrates, rather than a strict relationship with adhesive performance. Infrared spectroscopy of mucus revealed similar chemical signatures in both sucker and caudal peduncle mucus, suggesting that mucus composition does not change across the body to enhance adhesion. These findings indicate that goblet cell density and, thus, enhanced mucus production (rather than compositional changes) may aid the adhesive performance of climbing gobies, contributing to their ecological success. Understanding these adhesive mechanisms from tissue to whole‐animal levels of organization clarifies the specific factors that were specialized during the evolution of the distinctive locomotor behavior of these amphidromous fishes.

## Graphical abstract

Goby fishes may use their mouths and/or pelvic suckers to adhere and climb. In this study, we compared goblet cell density in the mouths and suckers of four goby species in two life stages and compared the chemical composition of mucus produced in the mouth versus the pelvic sucker. We found that that goblet cell density and, thus, enhanced mucus production (rather than compositional changes) may aid the adhesive performance of climbing gobies, contributing to their ecological success.

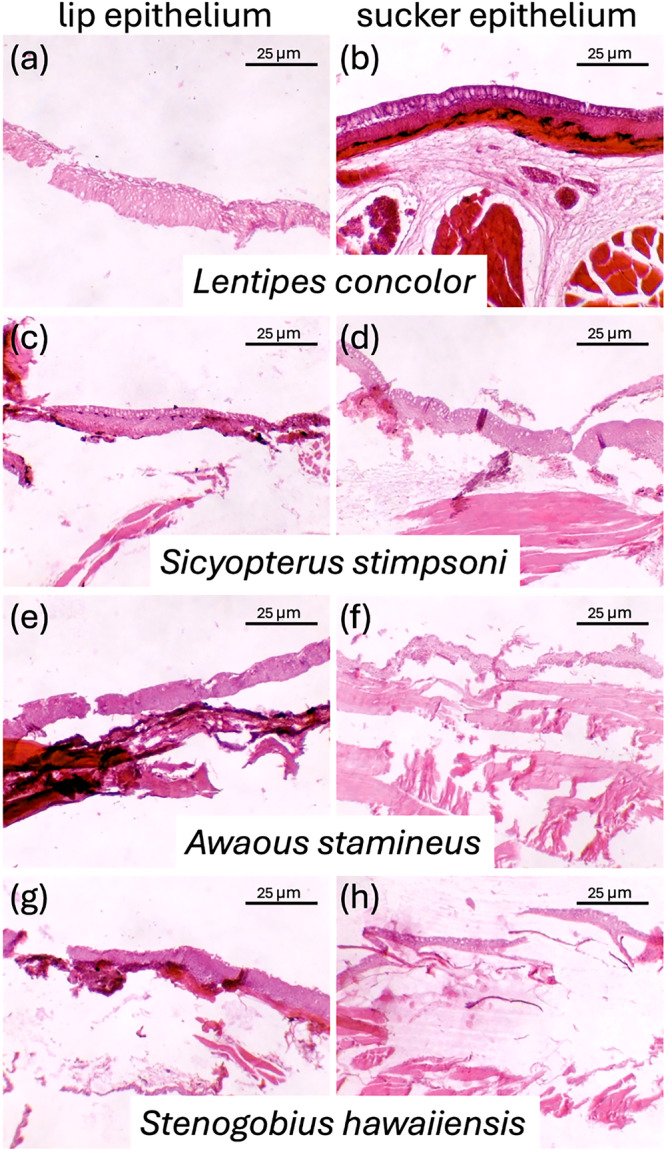

## 1 Introduction

Animals that migrate through challenging habitats provide an opportunity to examine the mechanisms through which traits contribute to functional performance during critical stages of the life cycle (Blob et al. 2006; Waterman 1999, 2001). By understanding such mechanisms, it may be possible to clarify the pathways through which adaptations arise that allow environmental challenges to be overcome. This study compares adhesive performance across several species of gobiid stream fishes (hereafter referred to as “gobies”), evaluating the contributions of both chemical and physical mechanisms to the ability of these fish to hold station in flowing water and, in some species, to climb waterfalls hundreds of meters tall. In this study, we test the extent to which levels of biological organization (e.g., anatomical structure vs. chemical composition) contribute to functional performance that influences the distribution of species in nature (Schoenfuss et al. 2013).

Many species of gobiid fishes that live in the streams of oceanic islands are amphidromous: adults live and breed in freshwater streams, with larvae that are swept into the ocean upon hatching from eggs and, after growing for several months, must migrate back to freshwater to return to adult breeding habitats (Schoenfuss and Blob 2007). Although some species remain in the lower stream reaches, others can climb tall waterfalls to reach upstream locations that, on many islands, are above the range of predators. Climbing behavior, and functions such as station holding in current, are enabled in gobies by the possession of a sucker that is formed by the fusion of the pelvic fins, which helps the fish to adhere to rock surfaces. However, species show considerable variation in sucker features that may correlate with adhesive strength and climbing ability (Palecek et al. 2021). For example, in the Hawaiian Islands, nonclimbing *Stenogobius hawaiiensis* possess large suckers that exert relatively low suction forces; in contrast, *Sicyopterus stimpsoni* can climb waterfalls over 100 m tall but have smaller suckers that exert suction forces capable of supporting 2.2x their body mass compared to *S. hawaiiensis*, in which the suckers can support ~1.5x their body mass (Maie et al. 2012). Other climbing species inhabit intermediate stream reaches between these taxa (e.g., *Awaous stamineus*). Current research suggests that adhesive ability is correlated with climbing ability, where species found at higher elevations in streams have stronger adhesive abilities (i.e., *S. hawaiiensis* < *A. stamineus* < *S. stimpsoni < Lentipes concolor*) (Palecek et al. 2020; Schoenfuss and Blob 2003, 2007).

Previous studies have proposed that differences in adhesive performance across goby species may relate to the muscle force applied dorsally on the fins as the fish is pressed to rock surfaces (Maie et al. 2012). This action enhances the vacuum inside the sucker, allowing higher surrounding ambient pressures to hold the fins (and fish) to the surface. However, adhesion can be accomplished in a variety of ways and may engage multiple components. For example, bats and echinoderms adhere to surfaces through a combination of suction and wet adhesion using mucus secretions from epithelial glands (Riskin and Fenton 2001; Sadeghi et al. 2012; Thomas and Hermans 1985). Mucus is a common epithelial secretion in fish that has known roles in protection against pathogens (Subramanian et al. 2008) and drag reduction during swimming (Daniel 1981), but its contribution to adhesive performance has received little attention (Wicaksono et al. 2016). Previous studies have demonstrated that the presence of mucus‐producing goblet cells correlates with the volume of mucus produced (Ma et al. 2018; Pflugfelder et al. 1997). In this study, we used histological and biochemical comparisons of stream goby species to test whether the density of mucus producing goblet cells and mucus composition, in addition to sucker size, contribute to variation in adhesive performance. If adhesion could be affected by biophysical interactions between the mucus of fishes and the substrate or surrounding water (Langowski et al. 2019; Thomas and Hermans 1985), factors such as water quality and pollution could have additional impacts on climbing ability and the sustainability of these species. Both the amount of mucus and its chemical composition could influence its contribution to successful adhesion (Ma et al. 2018; Pflugfelder et al. 1997; Wicaksono et al. 2016). In fact, some epidermal mucus excretions have chemistries that enhance adhesion (Langowski et al. 2019; Smith et al. 2019; Smith et al. 1999). Thus, understanding the role of mucus in adhesion is critical to understanding how these fishes accomplish their upstream migrations.

We hypothesized that greater goblet cell density, and therefore enhanced mucus production, increases adhesive performance in Hawaiian gobies. Consequently, we expect that goblet cell density is greater in species that are better climbers (i.e., have greater adhesive performance and penetrate further upstream), as well as in the morphological structures that directly adhere to substrates (i.e., suckers) compared to other parts of the body. We also hypothesized that mucus produced in the sucker may be chemically different from mucus on other epithelial surfaces. Mucus found in the pelvic suckers would likely contain components that attach to substrates with which a goby is likely to interact, thereby aiding adhesion (Wicaksono et al. 2016). This may indicate that the chemical composition of the mucus found on the sucker aids adhesion. To evaluate these hypotheses, we tested six predictions. (i) Species that use their mouths for climbing (inching climbers) will have greater goblet cell density in their lips than species that do not use their mouths for climbing. (ii) Goblet cell density in the lips of inching climbers will be greatest in juveniles when climbing ability is crucial compared to adults of the same species that rarely climb. (iii) Goblet cell density in the suckers of adults will correlate with climbing ability, wherein there will be a lower goblet cell density in the pelvic suckers of nonclimbers and pelvic sucker goblet cell density will increase with climbing ability. (iv) Goblet cells improve climbing ability and are not a function of age, so that juvenile climbers will have a greater goblet cell density in their pelvic suckers compared to their respective adults, but there will be no difference in goblet cell density of the pelvic suckers of nonclimbers across ontogeny. (v) Juvenile climbers will have greater goblet cell density in their suckers compared to their non‐climbing counterparts. (vi) Mucus will be different in its chemical composition across the body and sucker—in other words, the production of a specialized adhesive mucus will aid adhesion.

## 2 Methods

### 2.1 Specimen Collection

Five adults from four species (nonclimbing *Stenogobius hawaiiensis*, powerburst climbers *Awaous stamineus*, and *Lentipes concolor*, and inching climber *Sicyopterus stimpsoni*) were collected from their native streams in March 2020 on the Island of Hawai'i, using o'pae (prawn) nets. *S. hawaiianensis* and *A. stamineus* were collected from Waiakea Pond (Hilo, Island of Hawaii) using nets attached to long‐handled poles. *Sicyopterus stimpsoni* and *Lentipes concolor* were collected from Hakalau and Nanue streams (Hamakua Coast, Island of Hawaii) while snorkeling. Specimens from the latter two species were individuals that had successfully scaled a waterfall to reach adult breeding habitats. Juveniles of *S. hawaiiensis* and *S. stimpsoni* (five per species) were collected by dipnet below the first waterfall of Hakalau Stream. Consistent with prior studies, fish were maintained in aerated stream water with feeding rocks and housed at the Fisheries Research Station of the Hawai'i Division of Aquatic Resources in Hilo, Hawai'i, until killed for fixation in 10% buffered formalin (within 2–6 days of collection, depending on species). Fixed specimens were shipped overnight to home labs for further processing. Collections were conducted under Hawai'i Special Activity Permit 2021‐07, and all animal collection and care procedures were approved by the Institutional Animal Care and Use Committee (IACUC) at Clemson University (IACUC 2017‐085).

### 2.2 Histology

**Figure 1:**
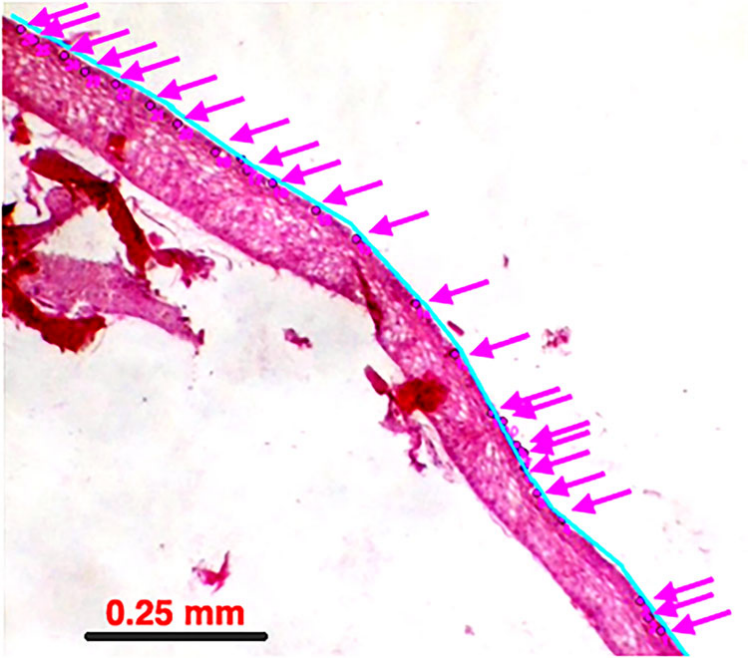
Representative tissue section analyzed in ImageJ software. The entire image white‐balanced and contrast was increased to aid analysis (Adobe Photoshop 26.7).

The pelvic sucker and lips were excised from each fish (10 *S. hawaiianensis* [5 adults, 5 juveniles], 5 *A. stamineus* [all adult], 10 *S. stimpsoni* [5 adults, 5 juveniles], and 5 *L. concolor* [all adult]) for histological study. Tissues were prepared following previously published methods (Cohen et al. 2020). Tissues were fixed for at least 1 week, dehydrated through a series of ethanol and xylene baths in a Leica automated tissue processor TP 1050 (Leica, Wetzlar, Germany), embedded in paraffin using a Thermo Scientific Microm EC 350 ± 1 embedding station (Waltham, MA), and sectioned with a stainless‐steel high‐profile blade at 6 µm thickness. Alternate cross‐sections were stained in a Leica Autostainer XL with hematoxylin and eosin (Y). Stained sections were captured using a 40X‐2500X LED Binocular Compound Microscope (AmScope, USA) and examined in ImageJ (Abràmoff et al. 2004), where epithelial distance was measured and where goblet cells were identified by location and number (Figure 1). Goblet cell density was assessed by counting the number of goblet cells found in a line of the epidermal tissue (density line) of interest using the multipoint tool. Tissue length was determined using the segmented lines tool after calibrating the tool using the scale bar incorporated with each slide.

**Figure 2:**
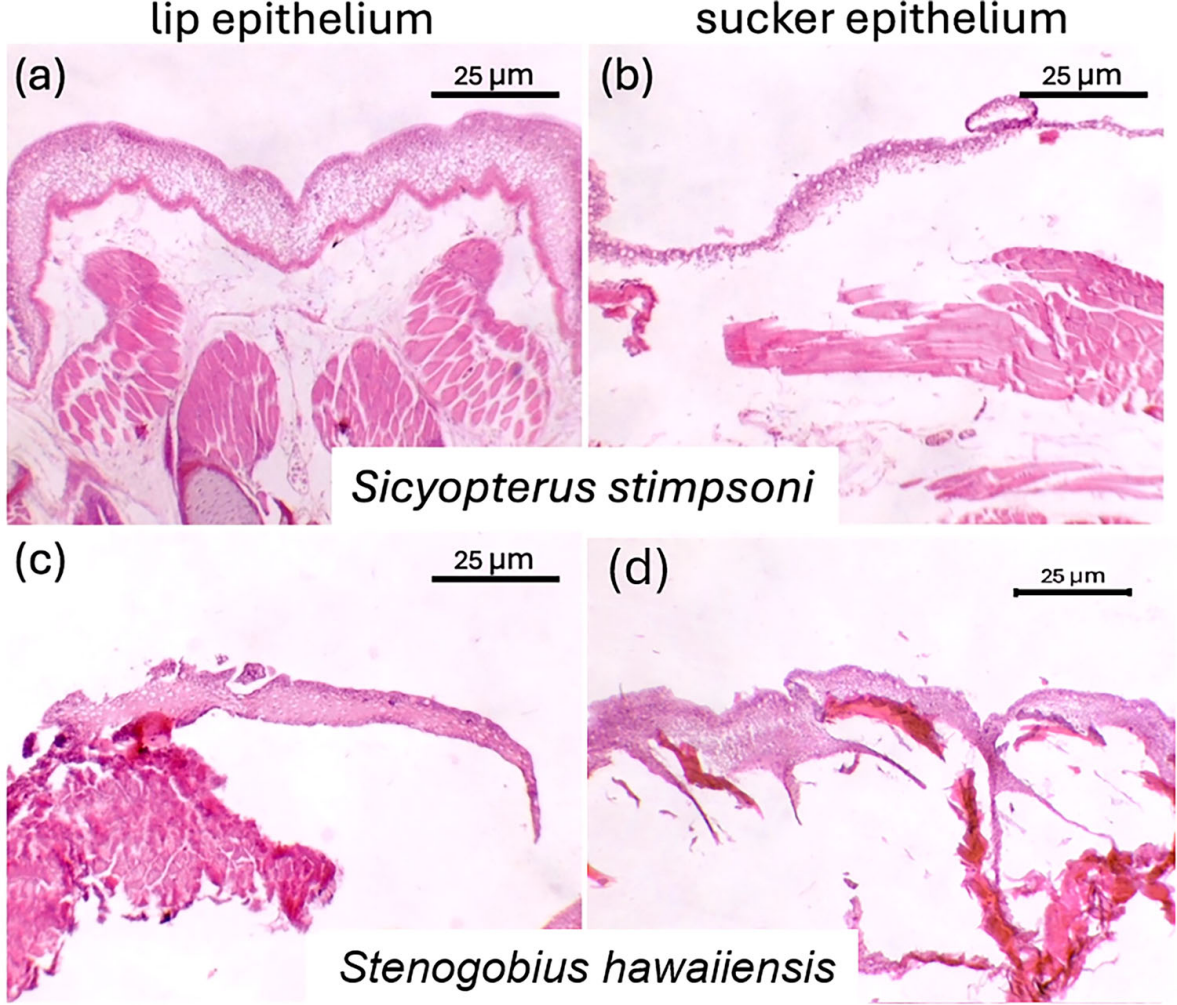
Representative histological images of juvenile lip tissue (left, a and c) and sucker tissue (right, b and d). *Sicyopterus stimpsoni* is shown in (a and b) and *Stenogobius hawaiiensis* is shown in (c and d). The entire image was white‐balanced and contrast was increased to aid analysis (Adobe Photoshop 26.7).

**Figure 3:**
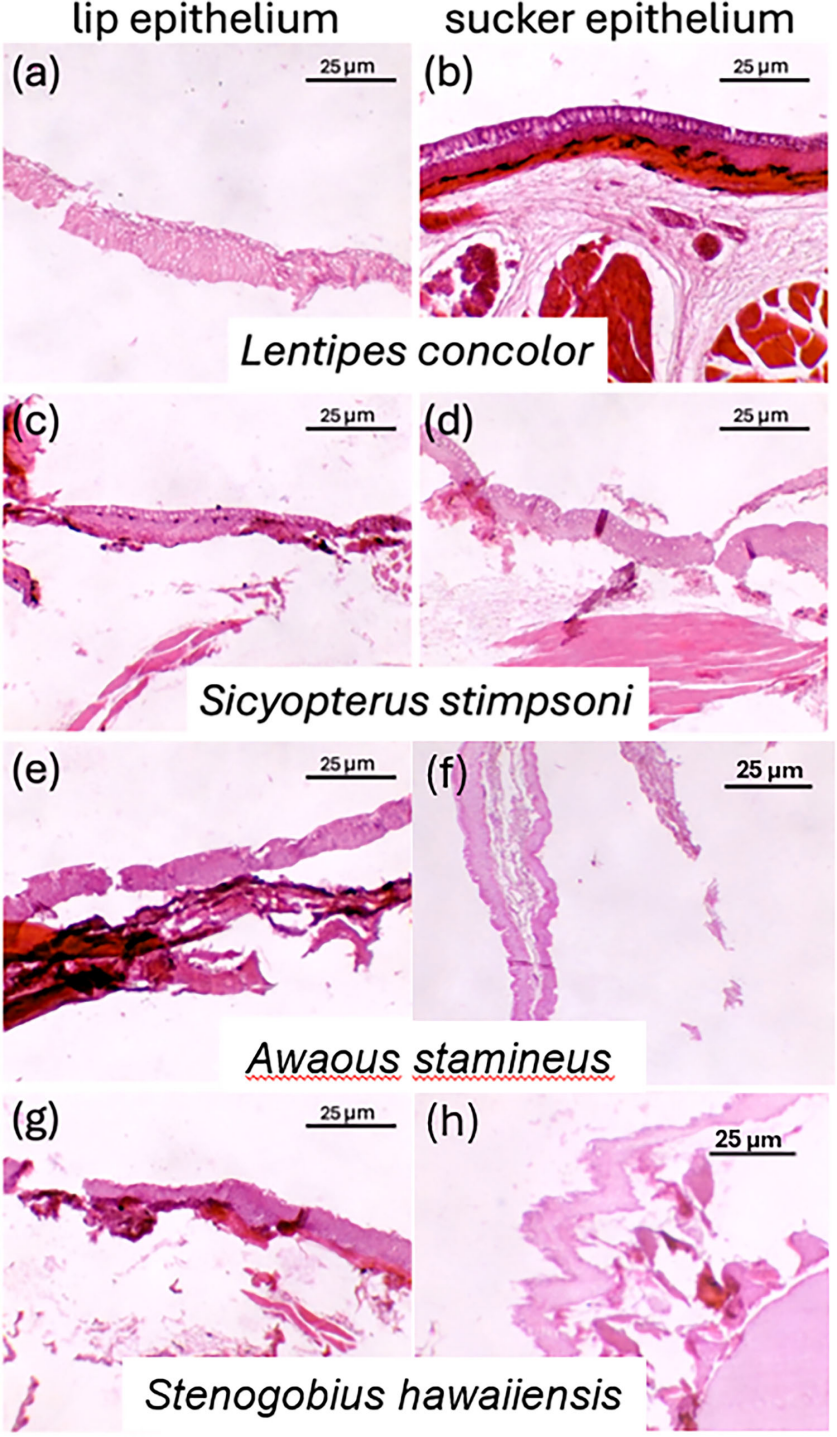
Representative histological images of adult lip tissue is presented on the left, histological images of adult sucker tissue is presented on the right. (a, b) From *Lentipes concolor*, (c, d) from *Sicyopterus stimpsoni*, (e, f) from *Awaous stamineus*, and (g, h) from *Stenogobius hawaiiensis*. The entire image white‐balanced and contrast was increased to aid analysis (Adobe Photoshop 26.7).

A total of 835 slides were collected from 35 fish. Of those slides, 158 sucker sections and 263 lip sections were of sufficient quality for evaluation. Slides that did not contain epidermal tissue (lost to washing/staining process) or had poor quality epidermal tissue (folded tissue, poorly stained tissue) were not imaged. Five hundred and twenty‐nine density lines of sucker tissue and 591 density lines of lips were analyzed. Example images of these tissues are displayed in Figures 2 and 3.

Quality control of histology data included monitoring trends to ensure that goblet cell density calculations did not gradually change as data were collected over time. In addition, 5% of the slides that included usable images were randomly reanalyzed blindly to ensure that results were duplicable. Reanalysis of the data indicated a strong correlation between the original and repeat observations (Pearson's correlation *r* ^2^ = 0.85; *p* < 0.01; 95% confidence interval: 0.71–0.92), consistent with expectations in medical pathology (Cross et al. 2000).

### 2.3 Spectroscopy

Because of limits to sample availability during the COVID‐19 pandemic (field work was conducted in March 2020), mucus was obtained from the caudal peduncle and the pelvic sucker of three specimens of a powerburst climbing species, *Sicyopus rubicundus*, available from a commercial supplier (Aquatic Arts, Indianapolis, IN).

The Fourier Transform Infrared Spectroscopy (FTIR) experiments were carried out using Nicolet iS50 spectrophotometer (Thermo Fisher Scientific, Walthem, MA, USA), in transmission mode using CaF_2_ window and deuterated triglycine sulfate detector. The sucker and caudal peduncle samples were collected on two separate CaF_2_ windows (diameter 12 mm and thickness 1 mm; EKSMA Optics, Vilnius, Lithuania). First a background scan was run using a clean CaF_2_ substrate, followed by running the mucus‐laden substrates. The scan size for samples was set to 128, and resolution was fixed at 4 cm^−1^.

Comparisons of peak area ratios were made between the mucus samples collected from the sucker and other epithelial surfaces to assess differences in chemical composition between mucus samples and to compare them with the existing literature (Chhatbar and Siddhanta 2015; Gao et al. 2016; Mecozzi et al. 2012; Sridhar et al. 2022; Suci and Geesey 1995; Wicaksono et al. 2016).

### 2.4 Statistical Analyses

A linear mixed effects model with specimen as a random effect was used to compare goblet cell density on the lips of nonclimbers, powerburst climbers, and inching climbers. A post hoc Least Means Squares test compared differences between groups. Linear mixed effect models with specimen as a random effect were also used to compare the lip goblet cell density between juvenile and adult *S. stimpsoni*, compare the sucker goblet cell density between juvenile *S. hawaiiensis* and *S. stimpsoni*, and compare the sucker goblet cell density across ontogeny in *S. hawaiiensis* and *S. stimpsoni*. To compare the sucker goblet cell density across all species, a Kruskal‐Wallis test using a Chi‐Square distribution was used. A post‐hoc Dunn's test using Bonferroni correction further explored differences between groups. All statistical analyses were completed in R (R version 4.0.5) using the lme4 and MuMIn packages.

## 3 Results

### 3.1 Histology

**Figure 4:**
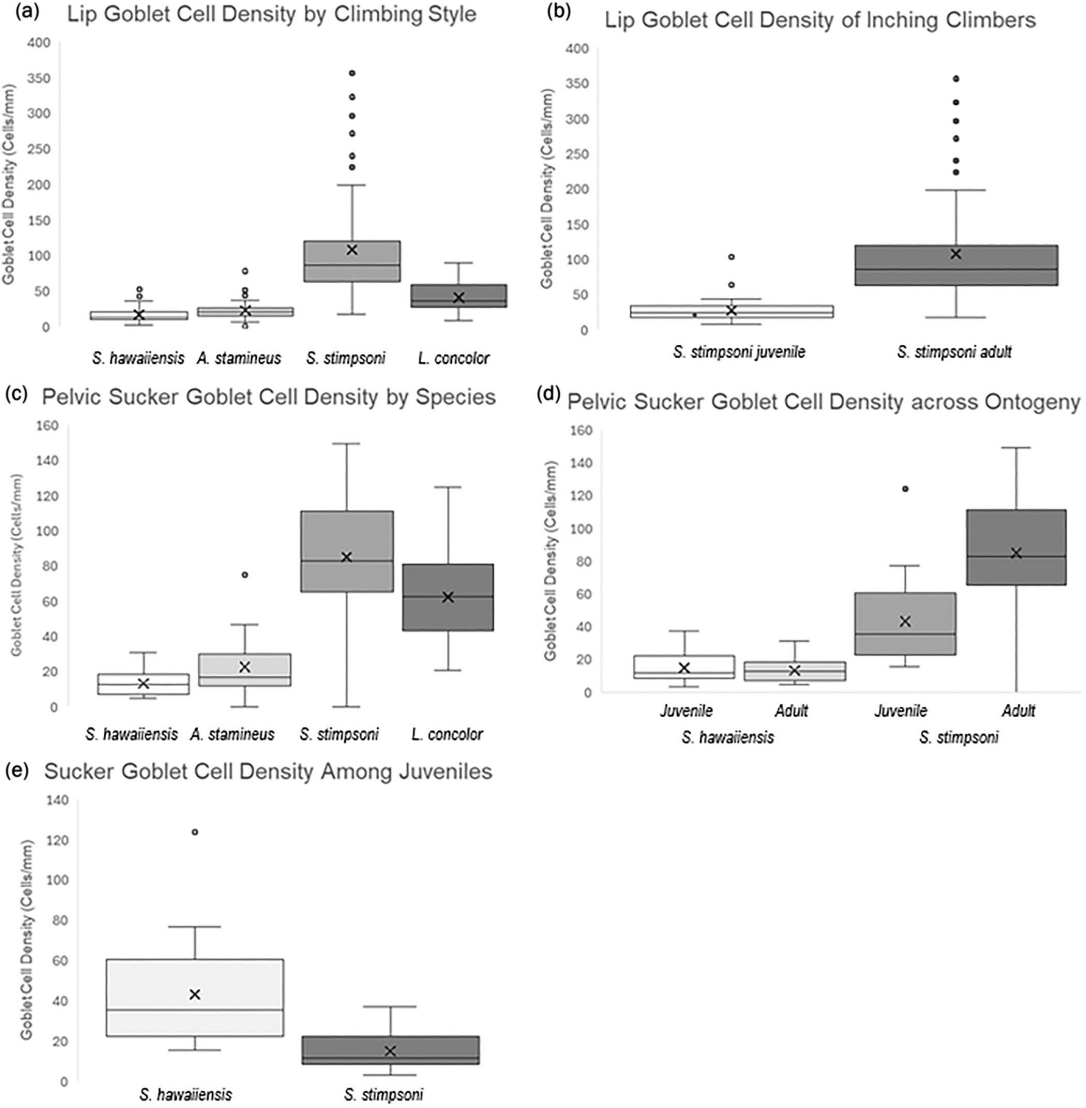
Average goblet cell density measurements across categories of amphidromous goby fishes. (a) Average goblet cell density of the lips across the three different groupings of climbing ability. (b) Average goblet cell density in the lips of *Sicyopterus stimpsoni* inching climbers across ontogeny. (c) Average goblet cell density of the suckers across the four Hawaiian species. (d) Average goblet cell density of the sucker across ontogeny for nonclimbing *S. Stenogobius hawaiiensis* and inching climbing *S. stimpsoni*. (e) Average goblet cell density of the suckers of juvenile nonclimbing *S. hawaiiensis* and juvenile inching climbing *S. stimpsoni*.

Goblet cell density on the lips of gobies was compared with the fishes grouped into three categories (Figure 4A): nonclimbing gobies (*S. hawaiiensis*, mean = 17.3 ± 2.04 standard error), powerburst climbers (*A. stamineus* and *L. concolor* mean = 27.5 ± 1.7) and inching climber (*S. stimpsoni* mean = 59.1 ± 6.4). Lip goblet cell density was three times greater in inching climbers than in non‐climbers (*p* = 0.04, *df* = 22.7, *t* = 2.6, SE = 16.2, EST = 42.2). In contrast, there was no significant difference between lip goblet cell density of non‐climbers and the powerburst climbers (*p* = 0.67, *df* = 22.1, *t* = −0.86, SE = 16.9, EST: −14.4) or between the lip goblet cell density powerburst climbers and the inching climbers (*p* = 0.19, *df* = 21.1, *t* = 1.8, SE = 15.3, EST = 27.8).

Average adult lip tissue in *S. stimpsoni* contained three times more goblet cells (90.4 ± 10.4) compared to the juvenile lip tissue (27.0 ± 2.6; *p* = 0.04, *df* = 8.4, *t* = −2.5, SE = 24.8, EST = −61.6) (Figure 4B).

Goblet cell density in the suckers (Figure 4C) was significantly greater in species with greater climbing ability (*S. hawaiiensis* mean = 13.0 ± 2.4; *A. stamineus* mean = 22.4 ± 3.5; *S. stimpsoni* mean = 80.7 ± 7.5; *L. concolor* mean = 62.2 ± 3.6, *p* < 0.01, *χ* ^2^ = 57.8, *DF* = 3). There was not a significant difference in the sucker goblet cell density of the nonclimbing goby *S. hawaiiensis* to the poor climber *A. stamineus* (*p* = 0.29, *z* = 1.1, SE = 10.8, CV = 28.6) or in the sucker goblet cell densities of the two best climbing species, *S. stimpsoni* and *L. concolor* (*p* = 0.11, *z* = 1.6, SE = 8.0, CV = 21.1). There was a significant difference in the sucker goblet cell density of the nonclimbing *S. hawaiiensis* compared to the best climbers, *S. stimpsoni* and *L. concolor* (*p* < 0.01, *z* = 5.6, SE = 11.2, CV = 29.6 and *p* < 0.01, *z* = 5.1, SE = 9.9, CV = 26, respectively). There was also a significant difference in the sucker goblet cell density of the poor climbing *A. stamineus* and the two best climbers, *S. stimpsoni* and *L. concolor* (*p* < 0.01, *z* = 5.6, SE = 9.2, CV = 24.2 and *p* < 0.01, *z* = 5.1, SE = 7.5, CV = 19.7, respectively).

Comparing sucker goblet cell densities across ontogeny (Figure 4D), there was no statistical difference between juveniles and adults in either *S. hawaiiensis* suckers (14.9 ± 2.1 vs. 15.7 ± 3.5, respectively; *p* = 0.65, *df* = 5.9, *t* = 0.5, SE = 4.4, EST = 2.1) or *S. stimpsoni* suckers (43.1 ± 5.3 vs. 80.7 ± 7.5; *p* = 0.07, *df *= 6.9, t = −2.1, SE = 13.9, EST = −29.2). Lastly, comparing the sucker tissue of juveniles in nonclimbing *S. hawaiiensis* (14.8 ± 2.1) versus the juveniles of the inching climber *S. stimpsoni* (Figure 4E), there was a three times greater goblet cell density in the *S. stimpsoni* suckers (43.1 ± 5.3; *p* < 0.01, *df* = 9.5, *t* = −3.2, SE = 8.8, EST = −28.5).

### 3.2 Spectroscopy

**Figure 5:**
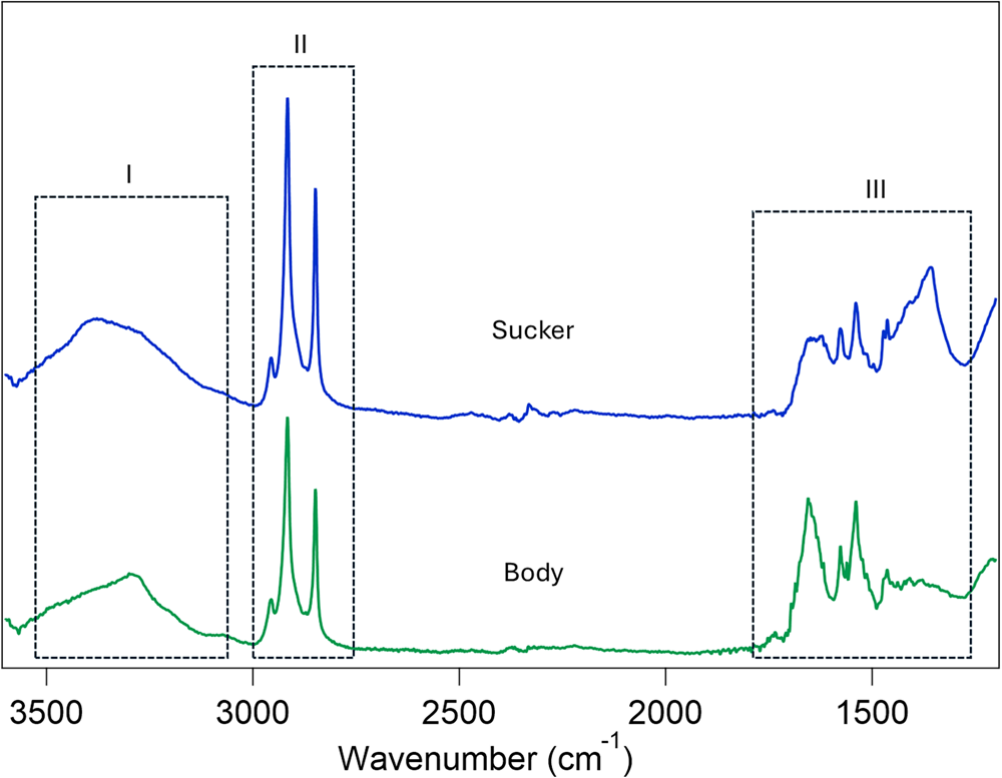
FTIR spectras of the mucus collected from the sucker (blue) and body (green) of *Sicyopus*.

The infrared spectroscopy analysis of mucus samples for the sucker and body showed peaks in three main regions (Figure 5, dotted rectangles). In the first region (I), there was a presence of a broad peak between 3000 and 3600 cm^−1^ for both sucker and caudal peduncle mucus The second region (II) encompasses three sharp peaks at 2954 cm^−1^, 2923 cm^−1^, and 2852 cm^−1^ that indicate stretching of alkyl groups that are CH_3_ (asymmetric), CH_2_ (asymmetric) and CH_2_ (symmetric) bonds respectively. Lastly, the third region (III) comprises multiple peaks between 1300 and 1700 cm^−1^. The key peaks in this region include 1740 cm^−1^ (carbonyl peak from the esters), 1650 cm^−1^ (amide I), and 1540 cm^−1^ (amide II) (Chae et al. 2022; Wicaksono et al. 2016). The region between 1200 and 1400 cm^−1^ (more pronounced in sucker mucus) may be associated with amide III signatures from the mucus proteins (Puray and Villaber 2023).

## 4 Discussion

### 4.1 Histology

When analyzing the mean goblet cell density of the goby lips, grouped by climbing style, several patterns emerge. As hypothesized, the lips of non‐climbing gobies had the lowest average goblet cell density, followed by the powerburst climbers. There was no significant difference between these two groups, independent of age, but both were significantly lower than the goblet cell density in the lips of inching climbers. In contrast to the nonclimbers and powerburst climbers, inching climbing gobies establish contact between their lips and the rock substrate during feeding and climbing (Blob et al. 2006, 2019; Cullen et al. 2013; Schoenfuss and Blob 2003). *S. stimpsoni* feeds by scraping diatoms from stream rocks (Cullen et al. 2013), and extra mucus produced by more abundant goblet cells may protect their lip epidermis against abrasion during feeding (Daniel 1981). Indeed, adults of this species possessed significantly greater mean goblet cell density in their lips than juveniles, which are just becoming herbivorous. These juveniles just recently underwent the metamorphosis of the position of the mouth from terminal to ventral position, corresponding to the transition to diatomaceous feeding (Schoenfuss 1997). While *S. stimpsoni* juveniles must survive the climb back to adult habitats, adult *S. stimpsoni* primarily use the oral sucker for station‐holding and feeding (Cullen et al. 2013). There may be a benefit for adult climbers to maintain goblet cell densities similar to con‐specific juveniles, as the stream reaches in which they live and breed are more turbulent compared to the gentler downstream habitats of nonclimbing species. This is supported by anecdotal evidence of adult upstream gobies repopulating upstream habitats within months of being displaced by a major hurricane (Fitzsimons and Nishimoto 1995). Alternatively, perhaps there is little to no pressure on adult climbers to maintain this greater goblet cell density from their juvenile stage. Previous studies have observed differences in climbing ability among these four species, with those species found at higher elevations demonstrating traits consistent with stronger climbing capabilities (Christy and Maie 2019; Maie and Blob 2021; Palecek et al. 2021). Aligning with these previous findings, the goblet cell density of the suckers of the four Hawaiian stream gobies generally appears to increase with increasing climbing ability. The non‐climbing estuarine *S. hawaiiensis*, and the lower stream reach inhabitant *A. stamineus*, did not differ in sucker goblet cell density, but had significantly lower sucker goblet cell densities than the strongest climbers, *S. stimpsoni* and *L. concolor*.

Examination of sucker goblet cell density across ontogeny shows that there is no significant difference between nonclimbing *S. hawaiiensis* juveniles and adults, nor was there a difference between inching climbing *S. stimpsoni* juveniles and adults. However, *S. stimpsoni* had significantly greater goblet cell density in the suckers compared to the non‐climbing *S. hawaiiensis* when pooling across ontogeny and when testing juveniles against each other. These results are congruent with the hypothesis that enhanced mucus secretion will aid in adhesive performance. Similar to previous studies on other adhesive species, it is likely that mucus promotes Stefan adhesion (Gu et al. 2016; Kappl et al. 2016; O'Donnell and Deban 2020; Wicaksono et al. 2016), where adhesion arises from the presence of a thin layer of fluid trapped between two surfaces, rather than acting as a bonding agent or glue (Balkenende et al. 2019; Von Byern et al. 2017; Crisp et al. 1985). This weak enhancement is essential when considering the biomechanics of climbing, as the constant attachment‐detachment cycle necessary to scale waterfalls would likely be costly if the fish had to fight against the attachment of a strong bonding fluid (Blob et al. 2006; Schoenfuss and Blob 2003; Smith 2002). Moreover, as for the lip during feeding, mucus may also play a protective role for the pelvic sucker during climbing (Mistri et al. 2018; Seo et al. 2020), as gobies must attach and detach hundreds of times to climb waterfalls, often on rough substrates (Blob et al. 2006, 2007; Schoenfuss and Blob 2003). Of course, goblet cells in fish epidermal epithelium are not limited to structures associated with climbing, and mucus deposited by goblet cells along the entire inferior epithelium of the fish may aid in wet adhesion, especially during inching climbing.

### 4.2 Spectroscopy

We characterized the epidermal mucus collected from the body and the pelvic sucker of two specimens of *S. rubicundus*, a powerburst climbing goby closely related to the Hawaiian species examined in the current study. Many of the resulting peaks were similar between areas of the body and comparable to those reported in the literature. One of the prominent peaks was found around 3390 cm^−1^. This region may be associated with the stretching vibration of amide (Amide A) and hydroxyl groups (Chae et al. 2022; Kumar et al. 2019; Puray and Villaber 2023; Sridhar et al. 2022; Faisal Tasleem et al. 2024; Wicaksono et al. 2016) and was identified as hyaluronic acid in a study on mudskippers (*Periophthalmus variabilis* and *Boleophthalmus boddarti*), two species of gobiid fishes that climb trees (*P. variabilis*) or that can lightly attach to a substrate on land but without climbing ability (*B. boddarti*) (Wicaksono et al. 2016). Hyaluronic acid is a glucosaminoglycan polysaccharide that may promote Van der Waals attraction or electrostatic attraction due to strong interaction energies between the hyaluronic acid and some of the substrates that gobies are likely to interact with as they adhere, such as silica and calcium carbonate (Wicaksono et al. 2016). Another peak found around 1645 cm^−1^ may indicate a carboxyl amide (Puray and Villaber 2023; Wicaksono et al. 2016; Yashwanthkumar et al. 2022). This functional group may induce hydrogen bonding, perhaps enhancing the consistency of the mucus (Gao et al. 2016) by providing cohesive forces to maintain a gel‐like matrix (Benhamed et al. 2014). The presence of amides may also indicate the presence of proteins similar to those of catfishes (Benhamed et al. 2014). Protein‐rich epidermal mucus likely plays a role in suppressing pathogens like bacteria and fungi (Mahadevan et al. 2019). Hydrocarbons responsible for four various peaks (alkanes at 2848 and 2918 cm^−1^) have also been previously found in the lipids of fish mucus samples from various species (Lewis 1970).

Interestingly, the same peaks that were found in the mucus samples collected from the body are present in the mucus samples that were collected from the sucker. This may indicate that the mucus is playing a role other than adhesion or in addition to adhesion. Indeed, epidermal mucus in fishes is known to play a range of roles (Benhamed et al. 2014; Daniel 1981; Eckes et al. 2008; Leonard et al. 2012; Reverter et al. 2018). While goby epidermal mucus may assist with adhesion, it is likely only contributing to weak Stefan adhesion when in contact with a substrate and the majority of the adhesive forces are due to suction (Wicaksono et al. 2016).

### 4.3 Broader Implications and Future Directions

Mucus serves a variety of functions in fishes, from providing immune functions, to mechanical protection, predator evasion, and drag reduction (Daniel 1981; Lauder et al. 2016; Mistri et al. 2018; Reverter et al. 2018; Subramanian et al. 2008). Mucus is complex in structure and function and not only offers insights into the fitness and adaptability of fish, but can inspire biomimetic gels that serve as antimicrobials, lubricants, and adhesives, especially those that work in wet or completely submerged conditions (Benhamed et al. 2014; Mahadevan et al. 2019; Mistri et al. 2018; Wicaksono et al. 2016). Adhesives have been developed following intense studies of mucosal secretions in other groups such as mollusks and amphibians (Deng et al. 2023; Hu et al. 2021; Zhang et al. 2019). The current study highlights the value of studying novel functions across levels of biological organization. The correlations between climbing ability and goblet cell density highlight the importance of these structures during locomotion and emphasize the contribution of multiple specializations to locomotor performance.

Future studies measuring the mechanical properties of goby mucus may uncover whether it can convey enhanced protection against friction in areas that are used for adhesion. While mucus among the climbing gobies may not be specialized for maximizing adhesion, it may be an exaptation, enhancing adhesive performance through improved Stefan adhesion as it protects epidermal tissue from repeated friction on rough substrates (Cullen et al. 2013; Gould and Vrba 1982; Wicaksono et al. 2016). Additionally, future studies should address not only the goblet cell density in epidermal tissue, but also the size of the goblet cells in epidermal tissue, as larger, more active goblet cells may produce more mucus while maintaining lower overall cell density in tissue. Stereological methods for morphometry may provide unbiased measures of goblet cell numbers and size, as is often used in biomedical research.

## 5 Conclusion

Goblet cell density was indeed greater in species with greater climbing ability and in structures directly involved in substrate adhesion, consistent with our predictions. Specifically, inching climbers that use their mouths to climb exhibited elevated goblet cell density in the lips compared to species that do not climb or use other climbing strategies. Contrary to expectations, adult inching climbers showed higher lip goblet cell density than juveniles, suggesting that mucus in this region may serve protective or multifunctional roles beyond climbing. In the pelvic sucker, goblet cell density correlated positively with climbing ability across species, supporting its role in adhesion. While ontogenetic comparisons revealed no significant change in sucker goblet cell density in nonclimbers, the prediction that juveniles would have greater density than adults in climbers was not supported. Nevertheless, juvenile climbers still exhibited higher sucker goblet cell density than juvenile non‐climbers, reinforcing the link between mucus production and climbing performance early in development. Finally, infrared spectroscopy revealed no substantial differences in mucus chemistry between sucker and body regions, indicating that regional biochemical specialization is unlikely to underlie adhesive success. Instead, these results suggest that the quantity of mucus production, rather than its composition, plays a primary role in supporting the adhesive capabilities and ecological success of these fishes.

## Author Contributions

**Amanda M. Palecek‐McClung:** conceptualization, formal analysis, data curation, writing original draft, visualization, methodology, investigation, editing. **Charles H. Christen:** formal analysis, visualization, methodology, investigation. **Dharamdeep Jain:** formal analysis, data curation, visualization, methodology, investigation, editing. **Ali Dhinojwala:** supervision, methodology, investigation, editing. **Richard W. Blob:** conceptualization, supervision, methodology, investigation, editing. **Heiko L. Schoenfuss:** conceptualization, supervision, methodology, investigation, editing.

## Conflicts of Interest

The authors declare no conflicts of interest.

## Peer Review

The peer review history for this article is available at https://www.webofscience.com/api/gateway/wos/peer-review/10.1002/jmor.70078.

## Supporting information

supmat.

clean juveniles.

clean mouthonly.

clean sicyonly.

clean sicysteno.

clean sicysucker.

clean steno.

gobygobletdata analysis.

MUCUS.

## Data Availability

The data is available as a supporting file.
